# Important Discussion

**Published:** 1886-05

**Authors:** 


AN INTERESTING AND IMPORTANT DISCUSSION.

We would call especial attention of our readers to the discussion on diphtheria and croup by the Galveston Medical club. It is one of the most interesting papers that has ever appeared in this journal, and will well repay a careful purusal, This progressive society is doing good work, and is to be congratulated on the deep interest the members take in its meetings and discussions. We commend this organization to the profession throughout the State as one worthy of imitation.

